# Production of the probiotic dessert containing sprouted quinoa milk and evaluation of physicochemical and microbial properties during storage

**DOI:** 10.1002/fsn3.3517

**Published:** 2023-07-21

**Authors:** Hanieh Yarabbi, Sahar Roshanak, Elnaz Milani

**Affiliations:** ^1^ Department of Food Science and Technology, Faculty of Agriculture Ferdowsi University of Mashhad Mashhad Iran; ^2^ Department of Food processing Iranian Academic Center for Education Culture and Research (ACECR) Mashhad Iran

**Keywords:** fermentation, hardness, non‐dairy dessert, probiotic, sprouted

## Abstract

One of the challenges of the food industry is detecting the potential of novel non‐dairy food matrices to deliver probiotic bacteria to humans as cholesterol‐free products, suitable for people with lactose intolerance and sensitivity to dairy proteins. In this study, the possibility of adding sprouted quinoa milk (SQM) at 0%, 50%, and 100% levels in probiotic non‐dairy dessert containing native *Lactobacillus plantarum* isolated from camel milk was investigated. Physicochemical, functional, microbiological, color, texture, and organoleptic characteristics of probiotic dessert samples were evaluated during 1, 7, and 14 days of storage at 4°C. According to the results, fat, protein, carbohydrates, and ash increased significantly during germination (*p* < .05). With boosting the SQM levels in the probiotic desserts, the number of soluble solids increased, and the syneresis decreased significantly (*p* < .05). The simultaneous increase in SQM levels and time caused an increase in acidity and decreased the moisture content of the samples. As the storage time increased, the intensity of the syneresis also decreased. The brightness index in all samples containing SQM was lower than in the control sample. During storage, the viable cell number of *Lactobacillus plantarum* in all samples decreased significantly. However, they were above the minimum required for FDA recommendation (6 log CFU g^−1^), varying from 4.6 × 108 CFU/mL to 4.3 × 107 CFU/mL for 50% SQM treatment. It was concluded that probiotic desserts containing SQM up to 50% could be properly presented in the market as gluten‐free and functional food products.

## Graphical abstract

This research was conducted to investigate the possibility of producing a probiotic dessert containing indigenous Lactobacillus plantarum isolated from camel milk and adding sprouted quinoa milk (SQM) at 0%, 50%, and 100% levels. The physicochemical, functional, microbiological, color, texture, and organoleptic properties of the dessert probiotic samples were evaluated for 1, 7, and 14 days of storage at 4°C. The results showed that probiotic desserts containing up to 50% SQM could be correctly presented on the market as functional, gluten‐free food products.

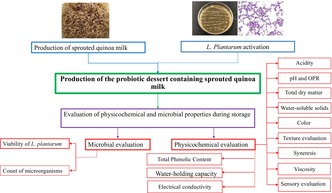

## 1 INTRODUCTION

According to the World Health Organization, functional food provides the body's basic nutritional needs. Cereals are important in global food production. Because they contain dietary fiber and prebiotics, protein, minerals, and vitamins needed by humans (Huang et al., 2022). Quinoa is one of the gluten‐free pseudo‐cereals with a relatively high nutritional value that can play an important role in the production of healthy foods (Jeske et al., 2018). Quinoa contains 8%–13% water, 64%–71% carbohydrates, 14%–17% protein, 4%–6% fat, and omega 3, 6, and 9 fatty acids. Quinoa has health benefits such as lowering postprandial glucose, lowering cholesterol, and lowering blood pressure (Dakhili et al., 2019). The good nutritional quality of quinoa is due to its high protein quality. Quinoa is often touted as a ‘superfood,’ it is exceptionally high in lysine. Quinoa seed is gluten‐free and rich in starch, so it is suitable for consumption by celiac patients. In terms of lipid content, it contains 52% unsaturated fatty acids, especially linoleic and 25% oleic. In addition, it contains tocopherols and carotenoids that have antioxidant and anti‐cancer activity (Maradini‐Filho et al, 2017). The presence of saponin in quinoa has caused its unpleasant, raw, and bitter taste and this phenomenon has led to the reduction of its use in food formulations. Therefore, performing simple pre‐processes treatments such as soaking and sprouting increases the nutritional value and improves quinoa's technological and sensory characteristics. Also, during the sprouting process, vitamins especially vitamin C, minerals, and proteins are greatly increased, but calories and carbohydrates are reduced convincingly (Singh et al, 2016). The addition of probiotic bacteria to the food matrices and keeping them alive during the food storage period is an important challenge. Dairy products are considered a good carrier for probiotic enrichment programs due to their high moisture, sugar, and protein content (Huang et al., 2022). Most probiotic products have a dairy base, but introducing a non‐dairy substrate for probiotic growth is another challenge. About 75% of the world's population suffers from lactose intolerance in milk, which is caused by a lack of lactase in the body. Some people also avoid animal products for various ethical or health reasons such as antibiotics, pesticides, and hormones injected into the cow. 2%–3% of children under the age of three are also allergic to cow's milk (Jeske et al., 2018). The aim of this project was to introduce novel plant‐based desserts as functional food. Therefore, the physicochemical and microbial properties of probiotic desserts containing native Lactobacillus plantarum and different concentrations of SQM as a desirable substitute for milk were investigated during 14 days' shelf life.

## 2 MATERIALS AND METHODS

### 2.1 Ingredients required

Sucrose and peeled quinoa (Sajima variety) were purchased from the local market, and skim milk powder was obtained from Iran Dairy Industries (Pegah). Also, gelatin, phenolphthalein reagent, and sulfuric acid were purchased from Merck, Germany. *Lactobacillus Plantarum* NIMBB003 registration code MT012188 isolated from camel milk as an active culture was prepared from Shams‐Bavaran Salamat Noor Company.

### 2.2 Method

#### 2.2.1 Sprouting of quinoa seeds

Quinoa seeds were soaked in water for 24 h. Then, it was placed in an incubator with 35% humidity at 21°C for 48 h until sprouts were produced (Fayyaz et al., 2018).

#### 2.2.2 Production of SQM

The sprouts were placed in water for 12 h and then heated at 80°C for 20 min. The aqueous extract of quinoa sprouts was separated (Fayyaz et al., 2018).

#### 2.2.3 Preparation of cultures

The lyophilized microorganisms were activated in MRS (De Man, Rogosa and Sharpe broth) and maintained at 80°C, in tubes containing MRS broth with the addition of glycerol (80:20). In each experiment, the cultures were activated and subcultured twice in 10 mL TSB (Triptic Soy broth) and incubated at 37°C for 24 h (Sabrina et al., 2014).

#### 2.2.4 Production of probiotic dessert

To produce a fermented dessert, 0%, 50%, and 100% SQM replaced skim milk powder (SMP) in the dessert formulation. The mixture of dry ingredients, including sugar and gelatin, was slowly added and placed at 40°C for 10 min to dehydrate the solid particles. Then, for pasteurization, the product was placed in a bain‐marie at 90°C for 10 min. After cooling down the dessert, native *L. plantarum* with a population of 10 log CFU g^−1^ was inoculated into the mixture and placed in an incubator for 150 min at 37°C. The final products were packed in individual plastic cups, sealed with a metallic cover, and stored at 4°C (Sabrina et al., 2014).

### 2.3 Physicochemical characterization

#### 2.3.1 The nutritional composition

Moisture, protein, fat, crude fiber, moisture, protein, fat, crude fiber, and ash of the raw materials containing white quinoa, sprouted white quinoa, and cow's milk were determined by standard methods (AOAC, 2005).

#### 2.3.2 Acidity

To measure the acidity (according to Dornick degree), 10 mL of the sample was taken and titrated in the presence of phenolphthalein with a gain of 0.1 normal until the appearance of a stable pink color (AOAC, 2005).

$$\begin{array}{c} \text{A milliliter of sodium hydroxide consumption}\times 10\\ =\;\text{acidity in degrees Dornic} \end{array}$$

#### 2.3.3 pH and oxidation–reduction potential (ORP)

The pH and ORP of dessert samples were measured using a pH meter model inolab pH 720 at 20°C during the 1st, 7th, and 14th days of shelf‐life according to the AOAC (2005).

#### 2.3.4 Water‐soluble solids (°Brix)

°Bx was measured at 20°C by a refractometer and expressed in grams per 100 g of a sample according to the AOAC (2005).

#### 2.3.5 Color

The color parameters (L*, a*, and b*) of dessert samples were detected using a HunterLab XE (Hunter AsSDSCiates Laboratory Inc.). Color was expressed as brightness (L*), redness (a*), and yellowness (b*). An average of four measurements was performed for each sample (Milani, & Koocheki, 2010).

#### 2.3.6 Texture analysis

The texture of the samples was evaluated using a texture analyzer model AMETEK Lloyd TA‐Plus Instruments Ltd. The samples of 20 × 20 × 20 mm were compressed up to 5% of the initial height. The penetration rate was 1 mm per second. The probe used was 100 Newtons (Sabrina et al., 2014).

#### 2.3.7 Syneresis

To measure the syneresis, 40 g of the dessert sample was centrifuged at 222 × g for 10 min at 4 ± 1°C. The clear liquid was discarded and the remaining compounds were weighed. Syneresis is expressed as the measured weight of the dessert to its initial weight as a percentage (Arioui et al., 2017).

#### 2.3.8 Water‐holding capacity

To measure the water‐holding capacity, 30 g of the sample was centrifuged with a speed of 13,500 × g for 30 min at 10°C. The released water was removed, and after the clot dries, the water‐holding capacity was obtained and the results were expressed as a percentage (Arioui et al., 2017).

$$\text{Water}-\text{holding capacity}=\left[\left(\text{weight of tube with clot}–\text{Empty tube weight}\right)/\text{initial sample weight}\right]\times 100$$

#### 2.3.9 Electrical conductivity measurement (EC)

EC was measured at specific time intervals by an EC meter (PQM I‐KOMBI INTEK). For this purpose, the electrode of the device was placed in the center of the dessert and after the number was fixed on the screen, the EC number (mS cm^−1^) was reported (Florek et al., 2007).

#### 2.3.10 Viscosity

The apparent viscosity of the desserts was measured by spindle SC4‐31 in shear rates (45 S^−1^) by means of a rotational viscometer (Anton PaarPhysica MCR30) at 25°C during 1, 7, and 14 days after production (Milani et al., 2011).

#### 2.3.11 Total phenolic content (TPC)

The total phenolic content was measured by the Folin–Ciocalteu method as described by Deng et al. (2018). The results were expressed as mg GAE g^−1^ of sample weight, where GAE stands for gallic acid equivalents.

#### 2.3.12 Sensory evaluation of dessert

The sensory properties of the product were evaluated by 12 trained individuals in terms of color, flavor, sweetness, hardness, and general acceptance on a 5‐point hedonic scale. In this test, the excellent sample scored 5, good 4, average 3, bad 2, and very bad 1.

#### 2.3.13 Statistical analysis

Chemical and microbial studies were carried out in three replications. Data were analyzed using SPSS version 17 (SPSS Inc.). Analysis of variance followed by Duncan's multiple range tests used to distinguish significant difference in treatments at *p* ≤ .05.

### 2.4 Microbiological characterization

#### 2.4.1 Viability of *L. plantarum*

It was cultured in an MRS agar medium after dilution and incubated in aerobic conditions at 37°C for 48 h (Milani et al., 2011).

#### 2.4.2 Count of microorganisms

Dessert samples were decimally diluted in sterile ringer solution. Then, 1 mL aliquots were poured into plates on MacConkey agar (Qlab). The results were reported as colony‐forming units per gram (log CFU g^−1^). The total counting of mesophilic microorganisms, coliforms counts, and also mold and yeast counts were performed according to Soni et al.'s (2020) report.

### 2.5 Experimental design and statistical analysis

A completely randomized factorial design was used to conduct the experiments. Independent variables include the amount of SQM (0%, 50%, and 100%) and storage periods (first, seventh, and fourteenth days) with three replicates. All data were statistically analyzed using Minitab 15 software. A comparison of mean data was done by Tukey's method.

## 3 RESULTS AND DISCUSSION

The physicochemical properties of white quinoa and sprouted quinoa are shown in Table 1. From the data, the content of 0.28% fat, 0.65% protein, 0.2% dietary fiber, and 0.26% ash increased significantly during germination.

**TABLE 1:** Physicochemical properties of raw materials.

Characteristic	Amount in 100 g white quinoa	Amount in 100 g sprouted white quinoa	Skim milk
humidity	9.01 ± 0.06^a^	8.96 ± 0.20^ab^	—
carbohydrate	61.70 ± 0.66^a^	60.93 ± 1.03^b^	4.52 ± 1.00
fiber	4.00 ± 0.41^b^	4.20 ± 0.53^a^	—
fat	4.92 ± 0.66^b^	5.20 ± 0.12^a^	3.25 ± 0.93
protein	14.99 ± 0.83^b^	15.64 ± 0.07^a^	3.22 ± 0.47
ash	4.55 ± 1.10^ab^	4.81 ± 0.07^a^	—
Saponin	0.05 ± 0.39	—	—

Table 1 presents the physicochemical properties of white quinoa and sprouted quinoa. Based on the data, the sprouting process significantly increased the amount of fat (0.28%), protein (0.65%), fiber (0.2%), and ash (0.26%). The decrease in dry weight, particularly carbohydrates, during germination is due to respiration. Enzymatic reactions leading to the breakdown of protein are necessary for plant growth, which could explain the boost in protein content during germination (Ikram et al., 2021). Lipids are metabolized to provide energy for protein biosynthesis, while structural lipids may improve, reflecting the new membrane formed (Fayyaz et al., 2018). The increment of ash content could be due to photosynthesis in sprouted quinoa, which synthesizes carbohydrates and absorbs minerals from the environment. Moreover, phytate reduction after germination has been observed to increase mineral bioavailability (Ikram et al., 2021; Pitzschke et al., 2015).

In pasta, Movahed et al. (2012) observed an increase in protein and ash by adding corn germ flour. Pitzschke et al. (2015) found that samples with high quinoa malt had more protein, ash, and vitamin B.

### 3.1 Changes in pH and acidity of probiotic desserts

**FIGURE 1:**
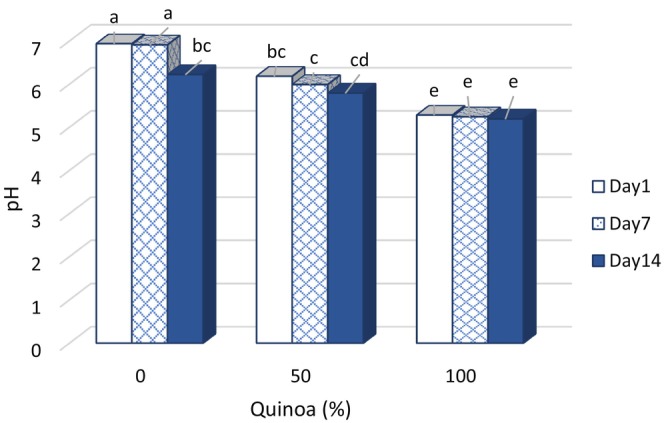
Changes of pH content in fermented desserts during storage at 4°C.

Increasing the amount of quinoa malt decreased the pH of the samples and increased their acidity (Figure 1 and Table 2). This is due to the germination process and the action of the enzymes alpha‐amylase, beta‐amylase (starch hydrolysis), protease, lipase, and lipoxygenase (lipid‐related enzymes), which degrade simple starch molecules. The protein matrix is also degraded to the secondary structure of the protein. During this phenomenon, the utilizable compounds of the probiotic organism as well as the activity of the microorganism increase, resulting in a significant increase in acidity (Soni et al., 2020).

**TABLE 2:** Changes in the acidity of the fermented dessert during refrigerated storage at 4°C.

The acidity of fermented desserts	1st day	7th day	14th day
100% of SMP	0.35 ± 0.09^c^	0.56 ± 0.44^c^	0.68 ± 0.17^c^
50% SMP: 50% SQM	0.43 ± 0.26^b^	0.7 ± 0.61^ab^	0.83 ± 0.05^b^
100 % SQM	0.48 ± 0.14^a^	0.74 ± 0.84^a^	0.98 ± 0.75^a^

With increasing storage time, pH decreased significantly and acidity increased. A significant difference in pH and acidity was observed in all fermented desserts during storage. It was also found that the fermented dessert containing SQM of *L. plantarum* had the highest acidity and the lowest pH (pH = 5.08) on the 14th day. The acidity of dairy products is influenced by the balance between nitrogen compounds from proteolytic reactions and lactic acid. Lactic acid is the result of the fermentative activity of lactic acid bacteria (Huang et al., 2022). Jeske et al. (2018) reported that the reason for the increase in organic acids during storage of fermented grain‐based beverages is the activity of lactic acid bacteria and the production of organic acids. Decomposition and consumption of nutrients by bacteria may be responsible for their growth and metabolic activity. *Lactobacillus plantarum* produces a wide range of organic acids such as lactic acid and acetic acid by forming four molecules of lactic acid from two molecules of lactose. It has been reported that a pH of about 3.5–4.5 in food formulations helps to increase the pH of the gastrointestinal tract, which improves the stability of probiotics. In addition, lactic acid provides a pleasant taste and prevents the growth of pathogenic microorganisms due to the increased acidity (Emma Ludena Urquizo et al., 2017). In the control sample, lactose is the only dominant sugar metabolized, but in samples containing SQM, sugar is metabolized by microbial flora in addition to lactose. The tendency for pH to decrease during storage is to be expected, which has been mentioned in most related studies (Milani et al., 2011; Soni et al., 2020). In addition, the higher sugar content in the fermented dessert formulation makes more monosaccharide sugars available to lactic acid bacteria, leading to their relative stimulation and lactic acid production. This is due to the high starch content of germinated quinoa. Enzyme activity during germination led to the formation of low‐molecular weight sugar compounds, and the content of sugar available to the organism during fermentation increased (Miranda‐Villa et al., 2019). This resulted in an increase in acidity and a decrease in pH compared to the control sample. In the study by Ayar (2013), the increase in sugar and fig content increased microbial activity and decreased pH. Mehdizadeh et al. (2021) studied the effects of different concentrations of date juice (0%, 50%, and 100%) on the rheological and sensory properties, as well as on the viability of probiotic bacteria in dairy desserts during storage at 5°C. The results showed that the pH of the samples decreased with increasing date juice content and inoculation with probiotic bacteria. Moreover, the pH showed a significant difference with increasing storage time.

### 3.2 Total dry matter of probiotic desserts

**FIGURE 2:**
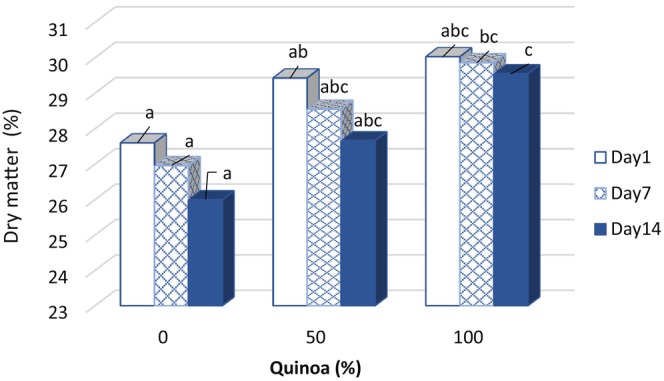
During storage at 4°C. Changes of the total dry matter content in fermented desserts during storage at 4°C.

The lowest and highest amounts of dry matter were found in the samples with 100% SMP and 100% SQM, respectively. The results showed that with the increase of SQM content in the formulation, the amount of dry matter in the samples increased significantly (*p* < .05). This was due to the higher dry matter content of SQM compared to SMP. Figure 2 shows the percentage changes in dry matter in samples containing 0%, 50%, and 100% SQM on the first, seventh, and fourteenth days after preparation. The highest percentage of total dry matter in the sample containing 100% SQM could be due to the water‐binding capacity of the quinoa starch. The starch becomes gelatinized during milk pasteurization and binds some of the water by preventing water evaporation, resulting in an increase in the total dry matter of desserts with 100% SQM.

In the study by Akkoyun and Arslan (2020), it was found that the protein content and dry matter of ayran increased with an increase in quinoa flour. Kundu et al. (2018) reported that milk blends prepared with a higher proportion of soy milk had progressively higher dry matter content, as soy milk contains higher total solids and protein content than almond milk. The results obtained in our research are in good agreement with the findings of Akkoyun and Arslan (2020).

### 3.3 Water‐soluble solids (°Brix) of probiotic dessert

**FIGURE 3:**
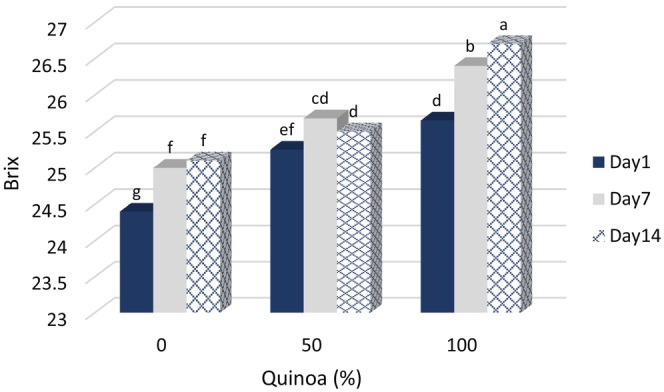
Changes of °Brix in fermented desserts during storage at 4°C.

Figure 3 shows the °Brix changes of the samples during storage. For all samples, °Bx increased with the increase in the percentage of SQM replacement. Our research results are consistent with the report of Ujiroghene et al. (2019), who reported an increase in total solids and °Bx in yogurt samples with a higher percentage of plant milk. The results of Furqani et al. (2017) showed that increasing the percentage of oat milk in yogurt decreased the pH and percentage of dry matter and significantly increased the acidity, protein content, and phenolic compounds of yogurt.

### 3.4 Color properties of the probiotic dessert

The L* factor, an indicator of brightness, was studied in all samples. The color brightness index in the fermented dessert formulation decreased with the increase of the amount of SQM and also with the increase of the storage time. The highest *L* * value in the control sample containing SMP resulted from the light scattering effect of the milk protein and fat particles (Chudy et al., 2020). The number of colorimetric components a* and b* was lower and higher, respectively, in the treatments with SQM than in the control sample. The color of the dessert became redder and yellower as the percentage of SQM increased (Table 3). The bright yellow color and the presence of betaxanthins and betacyanin in SQM could lead to an increase in the b* values of the samples (Escribano et al., 2017). Ayar (2013) indicated in the preparation of the traditional Turkish dessert with yogurt culture that the L* indices decreased with the increase of fig and sugar content. The results of our study are similar to the information reported in this research. According to the research of Ujiroghene et al. (2019), the changes in colorimetric components a* and b* are influenced by the degradation of reducing sugars, polyphenolic complexes, and enzyme activity during germination. Khalifa et al. (2020) studied the effects of adding quinoa flour at 1%–5% on the characteristics of low‐fat cheese. They found that the brightness of the samples decreased with increasing quinoa content. In addition, the b* indices increased, which was similar to the results of this study.

**TABLE 3:** Changes in the color components of the fermented dessert.

Fermented dessert formulation	L*	a*	b*
100% SMP	92.43 ± 0.29^a^	− 1 ± 0.60^a^	12.59 ± 0.45^c^
50% SMP: 50% SQM	89.51 ± 0.31^ab^	− 0.86 ± 0.04^b^	16.11 ± 0.63^b^
100% SQM	63.13 ± 0.29^b^	− 0.7 ± 1.07^c^	19.40 ± 0.11^a^

### 3.5 Hardness of probiotic dessert texture

**FIGURE 4:**
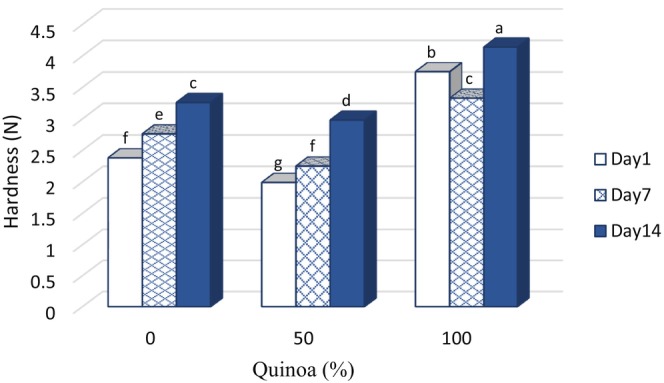
Changes of the hardness in fermented desserts during storage at 4°C.

The hardness of the dessert texture is considered a positive feature because it increases the mouthfeel and decreases the wateriness of the samples (Khalifa et al., 2020). For all samples, texture hardness increased significantly with the increase of SQM content and storage time of the samples (*p* < .05), but the interaction effect was not significant (Figure 4). The control sample with the lowest dry matter content had the lowest degree of hardness. In general, desserts with a high SQM content had a better texture than the other samples, so they performed better in the sensory evaluation.

As the percentage of dry matter increased, the hardness of the product texture increased. Much of the water in foods is linked by hydrogen bonds to factors such as hydroxyl groups in polysaccharides and amine and carbonyl groups in proteins (Hami & Goli, 2021). SQM is rich in proteins. The protein content of SQM increased the hardness of the samples through hydrogen bonding between amide hydroxyl and hydroxyl carbonyl groups with polar groups of other components of the dessert formulation (Arabi et al., 2018). In addition, hydrogen bonds are likely formed by electrostatic interactions between the charged groups of SQM protein and the charged part of gelatin (as an emulsifier and stabilizer with a negative charge). This factor may also be a reason for the increase in texture hardness in the presence of SQM (Khalifa et al., 2020).

Motamedzadegan et al. (2013) reported that gelatin interacts with milk protein as an enhancer of dry matter and basis of gel formation. This strengthens the structure and increases the viscosity and hardness of the tissue. Khalifa et al. (2020) studied the effects of adding quinoa flour in an amount of 1%–5% on the properties of low‐fat cheese. According to them, hardness increased with increasing quinoa content over time, which was comparable to the results of this study. The research results of Hami and Goli (2021) showed that the stickiness increased with increasing substitution of SQM with MMP. Quinoa flour retains and absorbs water molecules because it contains many hydroxyl groups in its structure. This phenomenon is due to the presence of water‐insoluble fibers, which allow stronger interaction with water through hydrogen bonding. Since quinoa flour has polar groups, it traps the water in the formulation in its structure and ultimately leads to an increase in the cohesion and stickiness of the ice. As a result, the water activity decreased and the consistency of the sample increased.

### 3.6 Synergistic effects of the probiotic dessert

**FIGURE 5:**
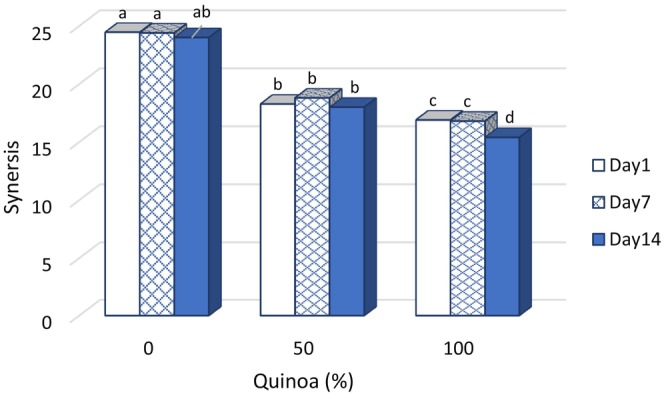
Changes of Syneresis in fermented desserts during storage at 4°C.

The results show that the highest syneresis was observed in the control sample with the lowest content of dry matter and hardness. This is because in this sample, the gel structure was weak. In the treatments containing SQM, syneresis decreased significantly with the increase of SQM content (Figure 5). The intensity of syneresis decreased with increasing duration of storage. The reason for this is the presence of fibers and proteins in the structure of quinoa, which have the ability to bind and retain moisture in their matrix. The structure of starch and sugars in SQ is an anionic compound, which probably has an increased water‐holding capacity due to the formation of bonds and interaction with positively charged milk proteins. The ability to link sucrose molecules with water molecules prevents the separation of free water (Arabi et al., 2018).

Syneresis decreased in samples with higher SQM content due to the presence of more simple sugars and amino groups. This phenomenon explains the increased water retention in these products (Arabi et al., 2018). However, the results of Marhamatizadeh et al.'s (2011) research showed that increasing the concentration of wheat malt extract increased the growth rate of probiotics and the consistency of yogurt. Therefore, the control sample had the lowest consistency while the yogurt with 6% barley malt had the highest consistency.

### 3.7 Water‐holding capacity of probiotic dessert

**FIGURE 6:**
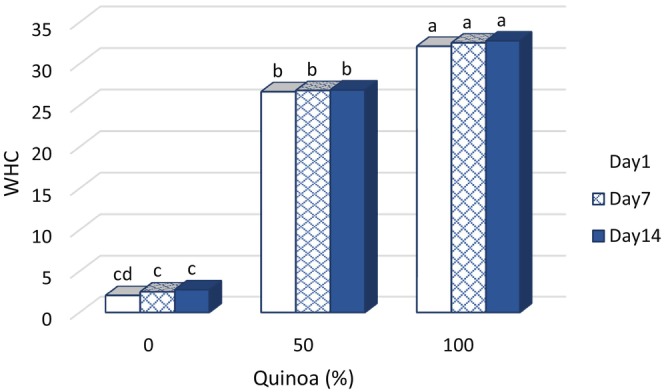
Changes of water‐holding capacity in fermented dessert during storage at 4°C.

Water‐holding capacity is an important parameter in the preparation of desserts because it is related to syneresis, which is due to the inherent instability of gels. The results show that the different SQM levels and time have a significant effect on the intensity of changes in water‐holding capacity, with *R* ^2^ being 97.5%. The control sample had the lowest water‐holding capacity. In this sample, the weak structure of the gel did not have a high ability to hold water. The total water‐holding capacity increased with time, which was due to the gradual evaporation from the surface of the dessert and the increase of solids. For treatments that contained SQM, the water‐holding capacity increased significantly with the increase in SQM content (Figure 6). The new balance between hydrophilicity and hydrophobicity created in these samples was able to affect the stability of the gel structure and reduce water loss (Khalifa et al., 2020). The increase in water retention is due to the addition of plant milk and the interaction of milk fat with plant protein. Amino acid composition, protein conformation, and the presence of polar and hydrophobic points on the surface of the protein structure increase water retention. Jeske et al. (2018) and Ujiroghene et al. (2019) reported an increase in water‐binding capacity in yogurt samples containing 50% and 100% germinated sorghum or SQM.

Water‐holding capacity is an important parameter in the production of desserts because it is related to syneresis, which is due to the inherent instability of gels. The water‐holding capacity of the dessert samples and their syneresis properties decreased with increasing SQM content. Figure 10a and Equation 1 show the relationship between water retention capacity and syneresis (in dessert samples).

(1) $$y=-1.1138x+25.416\;R^{2}=.8207$$

### 3.8 EC of probiotic dessert

**FIGURE 7:**
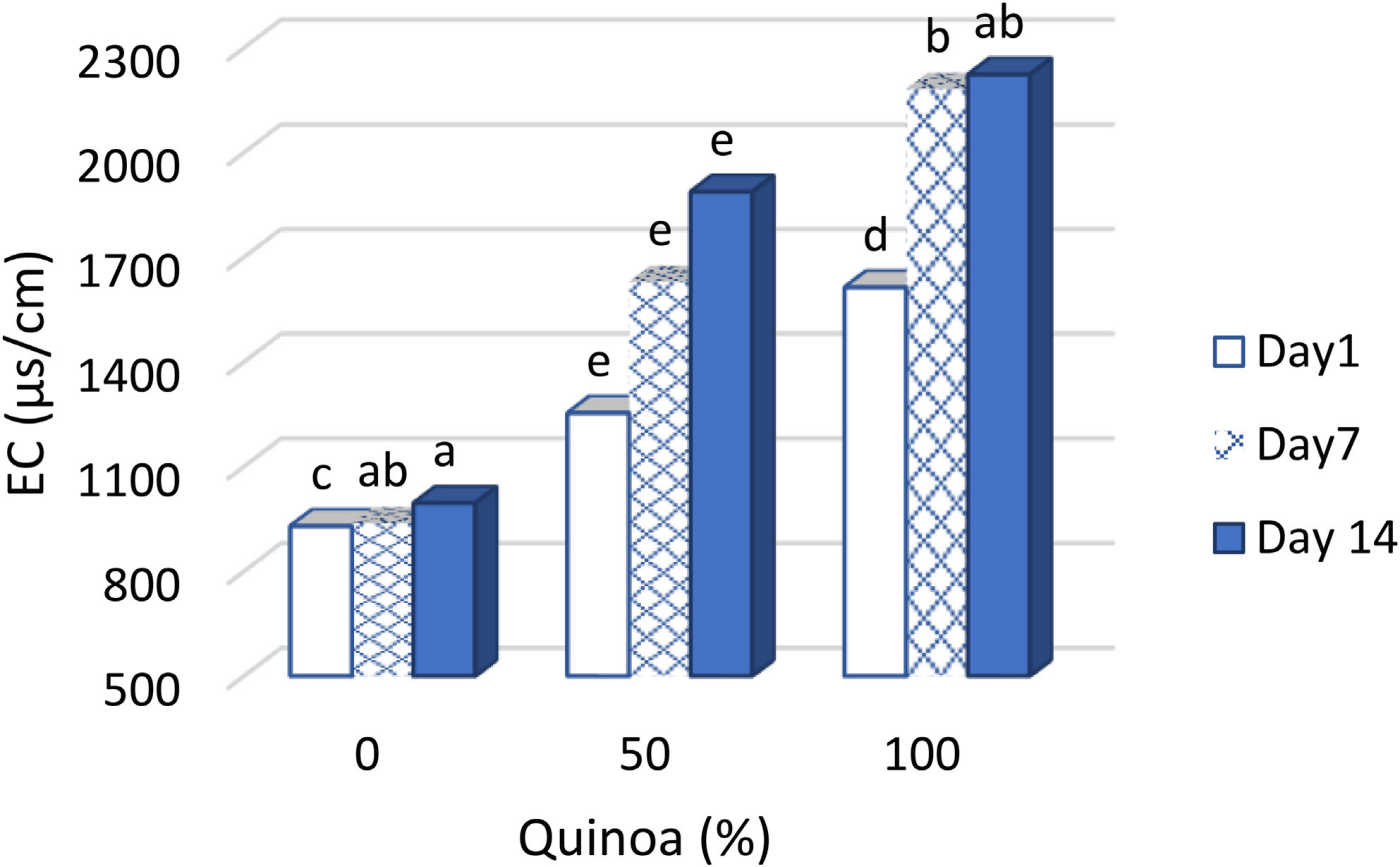
Changes of EC in fermented desserts during storage at 4°C.

The conductivity of a given material to electric current is referred to as EC. The total concentration of electrolytes can be measured by the conductivity value of the feed content. Milk quality is determined by the content of protein, fat, total solids (TS), non‐fat components (solids‐not‐fat, SNF), lactose, density, and acidity (Furqani et al., 2017). The researchers reported a direct relationship between the amount of EC in dairy products and the parameters of protein, TS, SNF, lactose, and mineral salts in the environment. Moreover, the value of EC significantly affects the value of density of dairy products (Banti, 2020). The value EC of the dessert with 100% SQM was higher than the other samples. This indicates the presence of mineral salts and other compounds in the samples with 100% SQM compared to other treatments. The lowest value of EC was observed in desserts with 100% SMP. In general, the amount of EC increased with increasing storage time (Figure 7). With the increase of SQM percentage in the treatments that contained SQM, the amount of water‐holding capacity increased significantly. As the SQM percentage increased in the treatments containing SQM, the water‐holding capacity increased significantly. Because the effect of protein content on water‐holding capacity and electrical conductivity was significant. As mentioned in Table 1, the protein content of SQM was four times higher than the protein content of SMP. The results of our study were in agreement with the reports of other researchers (Banti, 2020).

The EC of the food product determines the balance between water and food components, which is one of the problems in the industry. The conductivity measured in deserts is directly related to water‐holding capacity and vice versa with irrigation. Water‐holding capacity is one of the most important and measurable properties of desserts, and its reduction means a high economic loss for the industry and negatively affects the technological and sensory properties. The results show that as the water‐holding capacity in the dessert samples increased, so did the parameters EC and the concentration of total dissolved solids in the samples. Figure 10 shows the relationship between the water retention capacity and EC (10b) and the relationship between the degree of syneresis and EC (10c) of the desserts. The equations for these relationships are shown below:

(2) $$y=196.58x+695.42\;R^{2}=.8298$$

(3) $$y=-199.6x+2676.3\;R^{2}=.8554$$

### 3.9 Viscosity of probiotic dessert

The results of the analysis of variance table on the significant effect of quinoa or time on the viscosity changes showed that the viscosity in the control sample was lower than that in the samples with SQM due to the weak gel structure (Table 4). The binding of water to the components of SQM, especially to the proteins, increases the absorption rate of water and the stabilization of protein molecules. In addition, gelatin strengthens the gel structure by binding with the positive charges of the SQM proteins. During the shelf‐life, the mechanical strength of the gel also increased linearly with the increase in the concentration of SQM in the dessert formulation. This may be related to the high content of free carboxyl groups and the increase in the connection points with calcium and other groups. In this study, SQM was used as a substitute for milk due to its properties such as water‐holding capacity, emulsifying ability and stability. SQM contains a high total solids content, which affects the increase in viscosity. Similar results have been reported by other researchers for non‐dairy products (Arabi et al., 2018). According to Alkobeisi et al. (2022), quinoa starch gelatinizes during thermal processing of milk and absorbs water, leading to an improvement in the viscosity of the final products.

**TABLE 4:** Changes in the viscosity and TPC parameters of the fermented dessert.

Time period	Sample	Viscosity (mPa·s)	Total phenolic content (mg GAE/kg)
1st day	100% SMP	268.37 ± 1.20^i^	10.81 ± 0.49^d^
	50% SMP: 50% SQM	365.08 ± 0.29^f^	15.64 ± 0.42^b^
	100 % SQM	546.42 ± 0.51^e^	17.58 ± 0.56^a^
7th day	100% SMP	278.04 ± 0.11^h^	6.28 ± 1.09^g^
	50% SMP: 50% SQM	592.36 ± 0.32^d^	7.11 ± 0.38^e^
	100 % SQM	967.11 ± 0.78^b^	12.40 ± 0.50^c^
14th day	100% SMP	299 ± 1.01^g^	2.75 ± 0.18^i^
	50% SMP: 50% SQM	945.35 ± 0.64^c^	3.70 ± 0.74^h^
	100 % SQM	1166.92 ± 0.27^a^	6.5 ± 0.08^f^

### 3.10 Sensory evaluation of probiotic dessert

The parameters of color, taste, texture, and general acceptability of the different treatments were studied (Table 5).

**TABLE 5:** Evaluation score of sensory characteristics of fermented desserts during storage at 4°C.

Sensory features	Color	Aroma and taste	Texture	Mouthfeel	General acceptance
**Next day of production (Probiotic fermented dessert)**					
100% SMP	4^a^	3.75 ± 0.21^b^	4 ± 0.11^a^	3.7 ± 0.10^a^	3.5 ± 1.00^a^
50% SMP: 50% SQM	4 ± 0.07^a^	4 ± 0.17^a^	3 ± 0.19^c^	3.5 ± 0.66^a^	3 ± 0.69^a^
100% SQM	2 ± 0.09^b^	3 ± 0.34^c^	4 ± 0.69^a^	3.5 ± 0.82^a^	2.5 ± 0.31^b^
**7th day of production (Probiotic fermented dessert)**					
100% SMP	5 ± 0.55^a^	3.25 ± 0.66^a^	4.75 ± 0.65^a^	3.5^a^	3.5 ± 0.06^a^
50% SMP: 50% SQM	3.5 ± 0.12^bc^	3.5 ± 0.79^a^	4.25 ± 0.82^a^	3.25 ± 0.0^a^	3.5 ± 0.23^a^
100% SQM	2.5 ± 0.36^cd^	2.25 ± 0.20^cd^	4.5 ± 0.0^a^	3 ± 0.61^ab^	2.75 ± 0.04^ab^
**14th day of production (Probiotic fermented dessert)**					
100% SMP	4.125 ± 0.0^a^	2.375 ± 0.23^b^	2.5 ± 0.11^bc^	2.5 ± 0.4^ab^	3.15^a^
50% SMP: 50% SQM	3.25 ± 0.03^ab^	3.375 ± 0.39^a^	3.875 ± 0.06^a^	3.11 ± 0.15^a^	3.3 ± 0.15^a^
100% SQM	2.25 ± 0.98^bc^	2.15 ± 0.82^b^	4.25^a^	2.25 ± 0.08^ab^	2.75^ab^

The results show that the probiotic desserts have good texture and acceptability with increasing SQM content. The addition of more than 50% SQM significantly affected the color, flavor, and sweetness of the product. Thus, the aroma and flavor ratings decreased significantly with the increase of sprouted quinoa content due to the appearance of a new vegetable flavor. From the results, the control sample was rated very well and the treatments were adequately rated at 50% SQM. Thus, although there were slight differences in the acceptability of the control samples and the dessert samples treated with SQM, this did not result in significant changes in the samples with 50% replacement. The role of color in initial acceptance of products is very impressive (Ujiroghene et al., 2019). Bianchi et al. (2015) also reported that the taste score of the synbiotic fermented beverage prepared from SQM was about 2.5, which was due to the unusual taste of quinoa. Thus, the taste score decreased with the increase of SQM content. Therefore, natural flavoring agents should be used to increase the score of the products in their formulation. The high content of starch in quinoa preserves the matrix structure, consistency, and viscosity of the manufactured product. The gelatinization process of quinoa starch takes place at 55–65°C and is therefore considered an important factor in increasing consistency. Consumers particularly like the relatively firm texture and sweet milk taste (Dakhili et al., 2019). Jeske et al. (2018) reported that all plant fermentation products have high consistency. Khalifa et al. (2020) reported that the flavor and texture hardness of the product increased by increasing the quinoa content up to 50%. The highest values for overall acceptability and texture were found in the sample with 50% SQM. Quinoa has proven to be an emulsifier and acts as a water retaining and stabilizing agent (Khalifa et al., 2020). The research results of Huang et al. (2022) showed that the viscosity and consistency of the synbiotic beverage formulation increased by increasing the concentration of quinoa up to 70%. The sample containing 70% soy and 30% quinoa had the highest overall acceptance compared to the sample available in the market. Castro‐Alba et al. (2019) reported that the fermentation of quinoa flour successfully improved both the nutritional and sensory properties of the products.

### 3.11 Viability of native *L. plantarum* bacteria in probiotic dessert

The therapeutic effect of probiotic bacteria requires their viability and activity. Therefore, the number of live probiotic cells per gram or milliliter of the product is very important. The initial number of native probiotic bacteria was 4.2 × 108, and the results show that the highest number of *L. plantarum* in all treatments was reached on the next day of dessert preparation. The number of live cells of probiotic bacteria in all samples decreased significantly during storage. The results showed that the number of probiotic bacteria in desserts containing larger amounts of SQM was significantly (*p* < .05) lower during the storage period (Table 6). The reason for this phenomenon may be related to the presence of phenolic compounds in SQM, which inhibit the growth of probiotic bacteria (Bianchi et al., 2015). In this study, the number of *Lactobacillus plantarum* cells in the samples of 100% MMP and 50% MMP: 50% SQM was above 6 log CFU g^−1^ during the storage period, and the cell viability decreased to 7.43 log CFU g^−1^ at the end of storage. Bueno et al. (2014) showed that the number of starter bacteria and probiotic bacteria in yogurt containing strawberry, raspberry, and pitanga fruit pulp was lower than in control yogurt. Our results were similar to the report by Bianchi et al. (2015). Sabrina et al. (2014) reported that the presence of quinoa and its replacement with milk had no negative effects on the viability of *Bifidobacterium animalis* and *Lactobacillus acidophilus*. However, it is recommended as a desirable additive due to its health‐promoting properties. Alirezalu et al. (2016) also reported that due to the presence of phenolic compounds in blackberry and carrot yogurts, the number of *Streptococcus thermophilus* and *Lactobacillus bulgaricus* bacteria significantly decreased during the storage period (*p* < .05). Mehdizadeh et al. (2021) studied the effects of different concentrations of date juice as a sugar substitute on the quality characteristics of dairy dessert products.

**TABLE 6:** Results of counting the microbial population (Log CFU/g) of probiotic dessert.

Time period	Sample	*L. plantarum*	Coliform	Mold and yeast	Total count
1st day	100% SMP	7.95 ± 0.13^a^	ND	ND	4.91 ± 0.11^a^
	50% SMP: 50% SQM	7.46 ± 1.04^a^	ND	ND	4.44 ± 0.46^a^
	100% SQM	7.45 ± 0.46^a^	ND	ND	4.15 ± 0.70^b^
7th day	100% SMP	7.47 ± 0.39 ^ab^	ND	ND	4.44 ± 0.54^a^
	50% SMP: 50% SQM	7.12 ± 0.61^b^	ND	ND	3.90 ± 0.31^c^
	100% SQM	6.94 ± 0.58^c^	ND	ND	3.14 ± 0.97 ^d^
14th day	100% SMP	6.6 ± 0.93^c^	ND	ND	4.10 ± 0.82^e^
	50% SMP: 50% SQM	6.43 ± 0.16^d^	ND	ND	2.31 ± 0.12^f^
	100% SQM	5.33 ± 1.21^e^	ND	ND	2.52 ± 0.06^g^

Abbreviation: ND, Not detected.

Their results showed that the addition of more than 50% dates reduced the viability of probiotic bacteria due to the increase in osmotic pressure. Dates also contain large amounts of phenolic compounds that affect the viability of probiotic bacteria. Therefore, the number of probiotic cells decreased significantly during the storage period, but was within the standard range for probiotic products.

### 3.12 Counting of microorganisms

**FIGURE 8:**
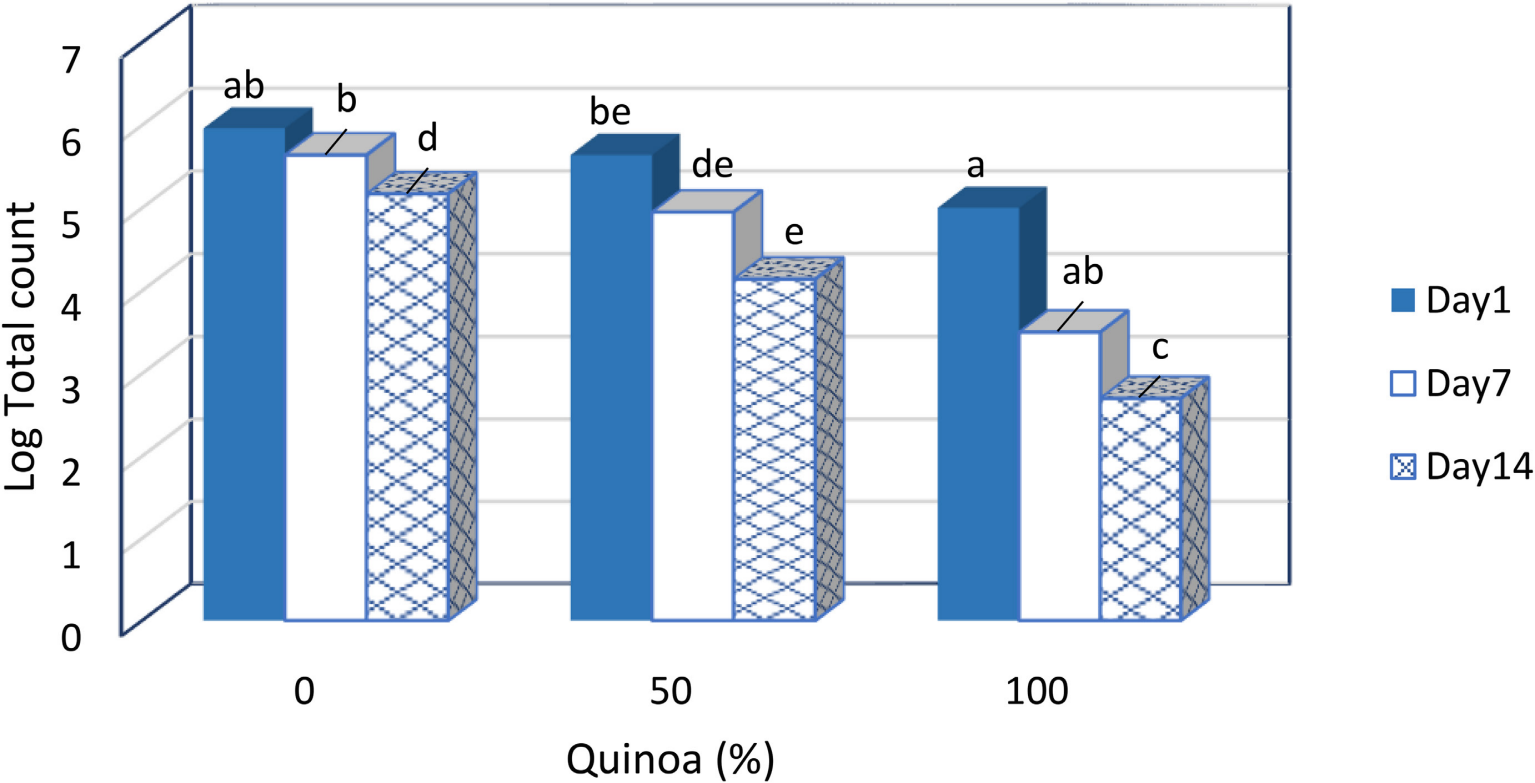
Changes Log total count of fermented desserts during storage at 4°C.

The results show that with the increase of SQM concentration, the total number of mesophilic bacteria increased significantly. As mentioned earlier, the presence of phenolic compounds and other antimicrobial compounds in SQM is probably one of the most important factors for the low total number of microorganisms during the storage period of desserts (Figure 8). In general, the total number of mesophilic bacteria in the dessert samples was within the standard limit and below 4 log CFU g^−1^. The absence of coliform bacteria, mold, and yeast in the samples indicates a proper pasteurization process and hygienic conditions during production and packaging (Table 6). It is considered microbiologically safe for human consumption.

### 3.13 Determination of phenolic compounds

Phenolic compounds have different nutritional and technological effects. The major phenolic compounds in SQM include flavonoids, catechin, vanillic acid, ferulic acid, kaempferol, and quercetin (Dakhili et al., 2019). The phenolic content of the samples ranged from 2.75 to 17.58 mg GAE g^−1^ during storage. Table 6 shows that the highest and lowest amounts of phenolic compounds were present in desserts with 100% SQM and 100% SMP, respectively. The results of the average data comparison showed that there is a significant difference (*p* < .05) between the different probiotic dessert treatments in terms of phenolic compounds content during the different storage days. The data also show that the phenolic compounds in the probiotic dessert samples decreased during storage at 4°C (*p* < .01). The results of our study show that the storage period significantly affects the phenolic content of probiotic desserts. This is because the phenolic compounds are not completely stable and decompose easily during storage. This can be attributed to the oxidation of the polyphenols. De Oliveira et al. (2017) showed that there was a decrease in all phenolic compounds in both sorghum grains and flour during the first 60 days of storage, and the retention of phenols ranged from 89.4% to 100% after 180 days of storage. Similar results were reported by Deng et al. (2018), who showed that the content of total phenols and procyanidins decreased by 20.2% and 24.2% at 4°C and 37.8% and 47.8%, respectively, at room temperature. Also, Zhang et al. (2021) reported that after 8 weeks, the total polyphenols and flavonoids content and antioxidant activity decreased in all whole wheat flours compared with week 0. TPC losses ranged from 16.39% to 20.88% and TFC losses ranged from 14.08% to 31.18%.

### 3.14 Oxidation–reduction potential

ORP means electron gain or loss and is considered an important factor in the quality control of milk fermentation products. This characteristic also reflects the metabolic activity of microorganisms (Ahmad et al., 2019). The results show that due to the decrease in the number of *L. plantarum*, the ORP parameter increased significantly during the storage period (Figure 10d). Several researchers consider the increase in the number of *L. plantarum* as a reason for the decrease of oxygen in the environment and the production of regenerating compounds due to cellular metabolism as one of the most important factors that decrease the ORP value of food (Morandi et al., 2016). Equation 4 shows the relationship between the number of probiotic bacteria and ORP.

(4) $$\text{OR}=16.888\;\mathrm{Log}\;L.\;\text{plantarum}+19.803\;R^{2}=.9721$$

**FIGURE 9:**
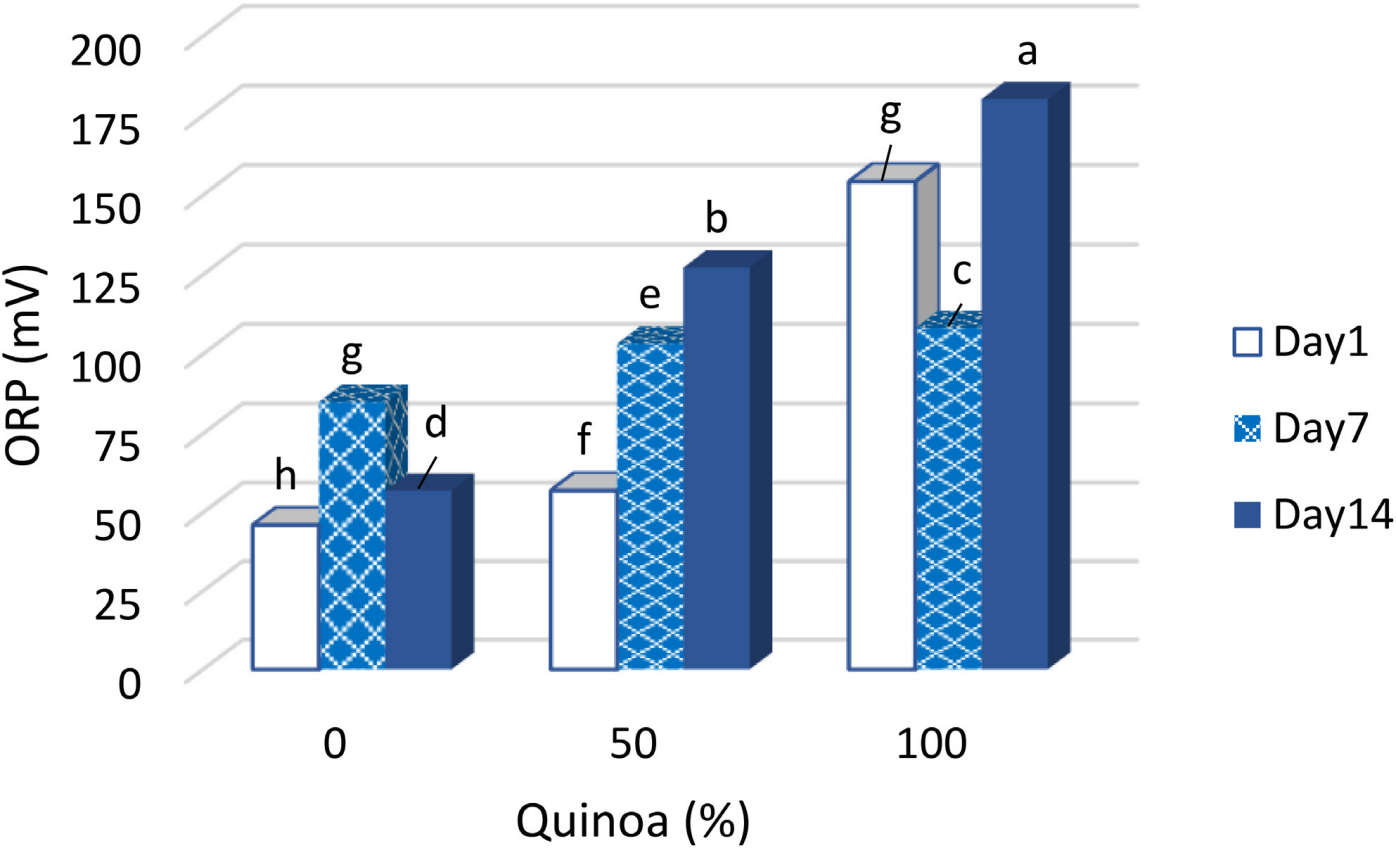
Changes of ORP contents in fermented desserts during storage at 4°C.

Figure 9 shows the changes in redox potential in the probiotic dessert samples during storage at 4°C. The ORP depends on the concentration of dissolved oxygen. The amount of dissolved oxygen in the final product depends on its initial amount in the starting medium, the entry of oxygen into the medium during production, and the penetration of oxygen during the storage period. The results of this study show that the ORP in the probiotic dessert samples increased during storage at 4°C (*p* < .01). The results showed that the highest number of *L. plantarum* and the lowest ORP value for all treatments occurred on the next day of dessert production. The number of live cells of probiotic bacteria and the amount of ORP significantly decreased and increased, respectively, in all samples during the storage period. This result is in complete agreement with the reports of Morandi et al. (2016). The lowest and highest ORP values were found in the samples with 100% SMP and 100% SQM on 14 (Figure 9).

**FIGURE 10:**
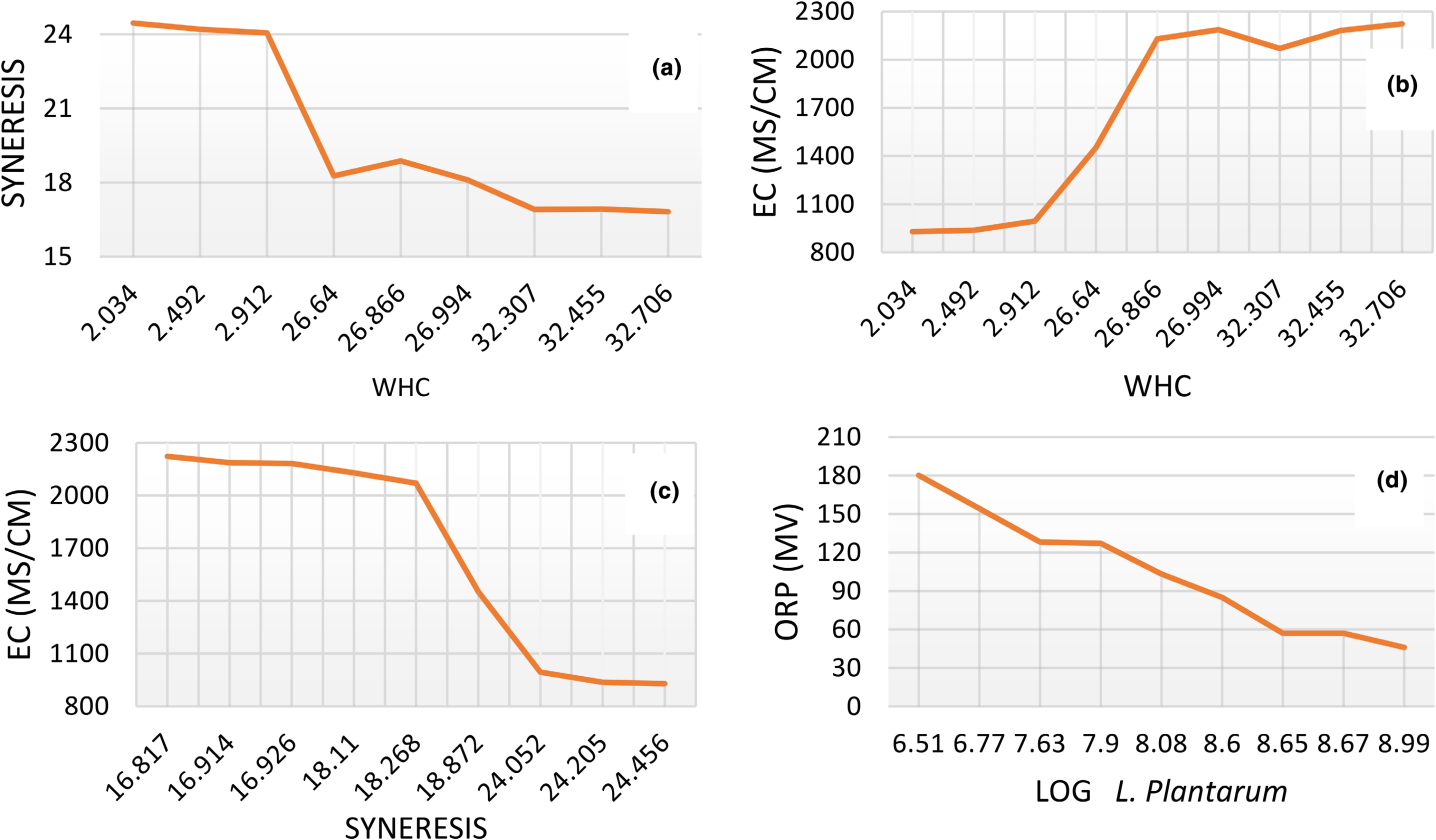
The relationship between WHC and syneresis (a), WHC and EC (b), syrensis and EC (c), *L. plantarum* count and ORP (d) in fermented dessert.

## 4 CONCLUSION

Nowadays, the production of functional food is increasing in many parts of the world. One method of producing these products is by using the capabilities of probiotic cells. While most fermented foods in the world are based on dairy products, less attention has been paid to the production of fermented products based on grains. Due to the sensitivity of some people to dairy products, as well as the increase in vegetarian diets among people, the demand for consuming probiotic plant products is rapidly increasing. Whole grains are a source of nutrients such as antioxidants, vitamins, minerals, and fiber. The addition of sprouted quinoa up to 50% to fermented desserts affects the physicochemical properties, and in most cases, better results were observed in viability, sensory properties, and synersis compared to the control and sample containing 100% SQM. Therefore, it seems that the fabrication of a novel fermented dessert containing native *Lactobacillus plantarum* and SQM as a functional food provides a new choice for consumers of plant‐based dairy alternatives. This product not only has a desirable taste with good nutritional values but can also be included in the diet of people suffering from diseases and genetic defects and food sensitivities such as lactose intolerance, celiac disease, and sensitivity to SMP protein. Consequently, the process of sprouting enhances the nutritional value of quinoa, making it a desirable ingredient in functional foods. By incorporating sprouted quinoa into fermented desserts, a plant‐based alternative with improved properties and benefits for consumers can be achieved. These findings suggest that adding sprouted quinoa to various food products could be a promising strategy to enhance their nutritional value and appeal to health conscious consumers.

## AUTHOR CONTRIBUTIONS

**Hanieh Yarabbi:** Formal analysis (equal); investigation (equal); software (supporting); writing – original draft (lead); **Sahar Roshanak:** Conceptualization (lead); methodology (lead); resources (lead); visualization (equal); writing – review and editing (equal). **Elnaz Milani:** Formal analysis (equal); methodology (equal); project administration (equal); software (lead); supervision (lead); writing –review and editing (lead).

## CONFLICT OF INTEREST STATEMENT

None declared.

## Data Availability

Our research data are available, and you can easily access the data in the supplementary section. Also, details of the data and how to request access are available by emailing the corresponding author. The authors confirm that the data supporting the findings of this study are available within this article's supplementary materials.
